# Association of admission and patient characteristics with quality of discharge letters: posthoc analysis of a retrospective study

**DOI:** 10.1186/s12913-017-2149-8

**Published:** 2017-03-21

**Authors:** Maaike Langelaan, Rebecca J. Baines, Martine C. de Bruijne, Cordula Wagner

**Affiliations:** 1 NIVEL, Netherlands Institute for Health Services Research, Otterstraat 118-124, 3513 CR Utrecht, The Netherlands; 2 Department of Public and Occupational Health & EMGO Institute for Health and Care Research, VU University Medical Center (VUmc), Amsterdam, The Netherlands

**Keywords:** Discharge letter, Quality of care, Patient safety, Hospitals, Retrospective study, Chart review

## Abstract

**Background:**

A complete, correct and timely discharge letter can communicate important information from the hospital to the general practitioner. The adequacy of the letter may vary with the patient and admission characteristics of the patient. Insight in the association between these characteristics and the presence and quality of the discharge letter will give rise to improvement activities for a better continuity of care after discharge.

The objective was to determine the presence, correctness and timeliness of admission information in discharge letters and to determine the association between patient and admission characteristics, including unplanned readmissions and the quality of the discharge letter.

**Methods:**

A post-hoc analysis of a two-staged retrospective patient record review study was performed in 4048 patient records in a random sample of 20 hospitals.

**Results:**

Nearly ten percent of the discharge letters are lacking in patient records in Dutch hospitals. In 59.1% of the discharge letters, one or more relevant components are missing. Important laboratory results, relevant information about consultations, answers to the questions of the referrer, changes in medication and follow up are often lacking. Discharge letters are more likely to be missing in elective patient admissions to a hospital, with a shorter length of stay, less comorbidity, and in readmissions. There was a significant variation in missing discharge letters between hospitals and between hospital departments.

**Conclusions:**

The quality of discharge letters varies with patient and admission characteristics.

## Background

Transitions from inpatient to outpatient care are considered a high risk process in patient safety terms. A discharge letter is intended to summarise events during hospitalisation, and prepare the outpatient physician or other referrer to resume care of the patient. A complete, correct and timely discharge letter can communicate important information back to the general practitioner or other involved outpatient health care workers, prevent adverse events and reduce readmissions to the hospital [1, 2]. Adverse events frequently occur in handover moments of care and could have been avoided in most cases. These adverse events might lead to readmissions and to extra costs [3]. A standardized discharge may decrease the number of postdischarge adverse events and rehospitalizations [1, 4]. Several studies found that discharge summaries often are not available or lack important information needed to help the general practitioner understand what should be done to avoid postdischarge adverse events. The review of Kripalani et al. showed that approximately 11% of the discharge letters and 25% of the discharge summaries never were received by the primary care physician [5]. Zegers et al. found that the discharge letter was unavailable in 13% of the reviewed patient records and more AEs were found in patient records with poor quality of the discharge letter [6].

All admissions may not be equally at risk for the absence or poor quality of the discharge letter. However, in-depth insight into specific types of admissions at risk is limited. Weiskopf et al. found that completeness of patient records varied with the underlying health status of the patient as sicker patients tend to have more complete records [7]. Research conducted on the association between admission characteristics and the presence and quality of the discharge letter will give rise to improvement activities for a better continuity of care from hospital to home. However, to our knowledge, no such research has ever been conducted.

The aim of this study was to assess the presence, correctness and timeliness of discharge letters in hospital records. Another aim was to determine the association between patient and admission characteristics, including unplanned readmissions, and the presence of the adverse events.

## Methods

### Design

The study design was a posthoc analysis of a retrospective patient record review study performed in a random sample of 20 Dutch hospitals: 4 university, 8 tertiary teaching and 8 general hospitals. From each hospital, a random selection of 100 admissions of discharged patients and 100 admissions of in-hospital deaths was made. Oversampling of deceased patients and patients admitted to a university hospital took place in order to gain sufficient data on these relatively small patient groups. During analysis, the results were corrected for this oversampling to make these representable for the total hospital population. Records from patients admitted to the psychiatry or obstetrics departments were excluded, as well as records from children aged less than one year. The study period was between April 1^st^, 2011 and March 31^st^, 2012. The design and methods of this study are described in more detail elsewhere [8, 9].

### Record review

The nursing and medical records of the admissions were reviewed by a team of 26 trained nurses and 10 trained physicians in a two-staged structured record review process. The reviewers never reviewed in hospitals where they had ever been employed. In the first stage, a nurse screened the patient records by using 16 screening criteria indicating potential adverse events, including unplanned readmissions. In this stage of the record review, the nurse assessed whether the discharge letter was present in the patient record and the date of sending. In the second stage, one physician reviewed the patient records with one or more positive screening criteria assessed at the first stage. Based on a standardized procedure the physician determined the presence of adverse events and their preventability. An adverse event was defined as an unintended injury among hospitalized patients that results in disability, death or prolonged hospital stay, and was caused by health care management [8]. In the records with at least one positive screening criterion for an adverse event the physicians judged also the correctness of applicable components of the discharge letter if available. Physicians were considered as the experts in judging the contents of the discharge letter. Therefore, the judgement of the correctness of the discharge letter took place at stage 2. In total 4048 patient records were screened by nurses and assessed for the presence and timeliness of the discharge letter, and 2632 patient records were reviewed by physicians and judged for the correctness of the applicable components of the letter.

Data were abstracted regarding the presence and correctness of the following components of the discharge letter: name of the patient, date of birth of the patient, date of admission, date of discharge, patient history, most important outcomes of tests, most important laboratory results, information about consultations and the conclusions, conclusions / diagnosis, answers to the questions of the general practitioner / referrer, treatment and prognosis, complications, treatment after discharge, changes in medication, follow up and appointments, and name and function of the discharging physician.

As official guidelines on components of discharge letters were not available and implemented in the Netherlands, the list of components was based on existing literature and one official guideline on information exchange between physicians and family practitioners [10, 11], expert opinions and unofficial guidelines for example from individual hospitals. Physicians also collected data about the timeliness of the discharge letter. Data about the admission characteristics (length of stay, admission department, urgency) were collected from the patient records and from the hospital administrative system.

### Statistical analysis

Descriptive statistics about the presence and components of the discharge letter were analysed using Stata 13.0 [12]. The relation between the presence and quality of the discharge letter and admission and patient characteristics was analysed with logistic multilevel regression analysis. Multilevel analysis was used because the data had a hierarchical structure: patients (level 1) were clustered within hospital departments (level 2) and hospital departments were clustered within hospitals (level 3) [13]. The 2^nd^ order PQL estimation procedure was used.

The presence of the discharge letter and the presence of discharge letter components were set as dichotomous (no/yes) outcome variables. For each component, separate regression analyses were run. The independent admission and patient characteristics were age, discharge status (deceased or discharged), admission urgency (elective/urgent), unplanned readmission (no/yes), one or more positive screening criteria for adverse events (no/yes), adverse event during admission (no/yes), preventable adverse event during admission (no/yes), and hospital type (university/tertiary teaching/general).

The variances of the model were tested for statistical significance using a one-sided Wald test.

ICCs were calculated for the hospitals and departments. The ICC indicates the relative influence of that level on the total variance of the outcome in a year. A high ICC at the hospital level means that there is less heterogeneity within hospitals and a high variation in the presence of the discharge letter between hospitals [13]. The variance at the patient level, the lowest level, is approximated by $\frac{\pi^2}{3}$ to calculate the total variance [13].

The multilevel analyses were carried out using MLwiN 2.30 and the application “runmlwin” in Stata 13.0 [14, 15].

## Results

A total of 4048 patient records were reviewed in 20 hospitals (Table 1). These were assessed for the presence of the discharge letter. The reviewers found that the discharge letter was not present in 500 (9.6%) of all patient records.

**Table 1:** Hospital and patient characteristics of the study sample

Hospital characteristics^a^	
Inpatient admissions (n)	4048
Hospital deaths n (%)	2025 (50.0)
Type of hospital n (%)	
University hospital	799 (19.7)
Tertiary teaching	1642 (40.6)
General	1607 (39.7)
Patient characteristics^a,b^	
Male sex %	50.2
Age (y), mean (SD)	60.5 (20.9)
Charlson index of comorbidity %	
Score > =3	3.7
Admission characteristics^a,b^	
Length of hospital stay (d), mean (SD/median)	6.3 (14.6/3)
Urgently admitted patients %	54.7
Admission is a readmission %	26.7
Admission is followed by an unplanned readmission %	17.7
Hospital departments %,	
Surgery	21.7
Cardiology	13.7
Internal medicine	18.7
Orthopaedics	11.2
Neurology	6.8
Lung diseases	6.9
Ear, nose and throat	3.6
Urology	4.9
Other	12.6
Presence of adverse events^a,b^	
One or more positive screening criteria in record n (%)	2632 (44.7)
Adverse events n (%)	390 (7.1)
Preventable adverse events n (%)	108 (1.6)

^a^ Patient admissions of obstetrics, psychiatry, <1 year and <24 h for non-deceased patients were excluded

^b^ Patient characteristics are weighted for overrepresentation of deceased patients and hospital type

Table 2 shows that discharge letters are more likely missing in patients electively admitted to a hospital, with a shorter length of stay, and in readmissions. Discharge letters of patients admitted to the neurology departments were more often missing than discharge letters of the cardiology departments. Patient records that were positive for one or more screening criteria had a lower odds for missing the discharge letter than records with all screening criteria negative. Although the association was not significant, the discharge letter was more often present in records with an adverse event (*p* = 0.07) and preventable adverse event (*p* = 0.36).

**Table 2:** Univariate multilevel analyses for the association between missing discharge letters and patient and admission characteristics adjusted for clustering at the hospital level and hospital department level

	OR for missing discharge letter (95% CI)
Type of hospital	
University	1.16 (0.57–2.35)
Tertiary teaching	1.21 (0.67–2.17)
General	RC
Admission department	
Cardiology	RC
Surgery	0.96 (0.59–1.58)
Internal medicine	1.00 (0.64–1.58)
Ear, nose, throat	1.67 (0.82–3.40)
Lung diseases	0.78 (0.45–1.35)
Neurology	0.31 (0.15–0.62)**
Orthopaedics	1.18 (0.66–2.10)
Urology	0.72 (0.34–1.54)
Other	0.52 (0.31–0.85)**
Age cat	
1–18 years	1.02 (0.55–1.88)
19–40 years	1.32 (0.85–2.03)
41–65 years	1.05 (0.79–1.39)
66–79 years	1.19 (0.91–1.55)
>80 years	RC
Urgently admitted patients	0.70 (0.56–0.87)**
Patients deceased during admission	0.81 (0.66–1.00)
Admission is a readmission	1.30 (1.05–1.60)*
Admission is followed by an unplanned readmission	0.89 (0.61–1.30)
Admission to a non-surgical unit	0.81 (0.60–1.09)
Length of hospital stay	0.99 (0.98–1.00)*
Charlson index of comorbidity	
Score > =3	1.35 (0.94–1.94)
One or more positive screening criteria	0.67 (0.54–0.83)**
Adverse event	0.70 (0.48–1.02)
Potential preventable adverse event	0.73 (0.37–1.44)

* p < 0.05

** p < 0.01

After correction for covariates the intercept variation for the hospital level and the department level was statistically significant (*p* < 0.05). This implies a significant variation in missing discharge letters between hospitals and between hospital departments. The clustering of missing discharge letters in departments (ICC 9.4%) was about one and a half times higher than in hospitals (ICC 6.7%).

In 2632 patient records at least one positive screening criterion for containing an AE was found. These records were judged the correctness of different components of the discharge letter. In 59.1% of the discharge letters reviewed by the physician, one or more applicable components were missing. Table 3 shows the absences and quality of discharge letter components. Important laboratory results, relevant information about consultations, answers to the questions of the referrer, changes in medication and follow up are often lacking.

**Table 3:** Absence and inadequacy of applicable discharge letter components

Discharge letter components	Absence of applicable components (*N* = 2632) (%)	Component present but inadequate (% of present components)
Name, address and function of receiver	1.4	-
Name and function of receivers of a copy of the letter	34.2	-
Name and date of birth of the patient	0.7	0.4
Address of the patient	0.9	-
Date of admission	1.5	-
Date of discharge or death	2.0	-
Patient history	7.1	3.8
Most important outcomes of tests	6.5	2.8
Most important laboratory results	31.6	1.6
Information about consultations and the conclusions	21.1	4.3
Conclusions / Diagnosis	2.2	3.5
Answers to the questions of the general practitioner / referrer	11.7	0.5
Treatment and prognosis	3.1	3.0
Complications	9.7	3.1
Treatment after discharge	7.1	0.7
Changes in medication	15.2	0.9
Follow up and appointments	10.8	-
Name and function of discharging physician	0.3	-

- Not applicable

The median of the duration of sending the discharge letter after discharge was 7 days (IQ range 1 day – 21 days). 13.6% of all letters did not seem to reach the general practitioner within 31 days after discharge.

In 373 admissions, the admission was followed by an unplanned readmission of which 153 (41.1%) readmissions were within 30 days after discharge. 35 (22,9%) of these patients were readmitted within 3 days after discharge. In 107 of 153 (69.7%) of the unplanned readmissions within 30 days, the discharge letter of the index admission was missing or incomplete at the time of reviewing and consequently at the time of readmission. In patients who were not readmitted to the hospital 45.5% of the letters were missing or incomplete.

## Discussion

Our study shows that 9.6% of the discharge letters are lacking in patient records in Dutch hospitals. Discharge letters that are available are often lacking certain components. Just as found in other studies on the completeness of patient record data we found that some components of the discharge letter were more often complete than others [16]. Information on name, address and age of the patient is complete for 98% of the patients. Data on receivers of the letter, relevant laboratory results, consultations and changes in medication are currently only available in 65 to 85% of the patients. If the components are available, our data shows that the information is often correct. Besides the completeness and correctness of the discharge letter, it is of importance that the letter is sent within a reasonable time.

The study also gives an insight into groups with a higher risk of missing discharge letters. Especially patients who are electively admitted, with a shorter length of stay, or readmissions are vulnerable to missing discharge letters. On the other side, discharge letters are more often available in patients with less comorbidity. This is not in concordance with findings in other studies that showed that sick patients have more complete electronic patient records [7]. It also seems that patients who experience an undesirable incident, i.e. an adverse event or experienced one or more positive screenings criteria, are more likely to have a discharge letter sent to their referrer, mainly the general practitioner.

One of the reasons of the incompleteness, incorrectness and untimeliness of the discharge letters may be the lack of an adequate standard outlining the components that each discharge letter should contain and guidelines on the timeliness of sending the discharge letter. Another reason may be a lack of a structural implementation of writing discharge letters in the medical curriculum. An adapted masters education program and a fast electronic sending system of the discharge letter to the general practitioner may decrease the duration of sending the letter [17].

The rate of missing discharge letters varied significantly between hospitals and between hospital departments. Moreover, the variance on the department level was almost one and a half times higher than the variance on hospital level. This implies that there is more room for improvement in the availability of discharge letters at the department level than at hospital level. The differences between hospitals and departments may be caused by differences in knowledge, dissemination or implementation of communication guidelines, such as the guideline of the Dutch College of General Practitioners or the standard of the Joint Commission on the Accreditation of Healthcare Organizations [10, 11]. Implementation of these guidelines is expected to improve the communication between medical specialists and general practitioners. To monitor the implementation of the guideline and the quality of discharge letters audits and feedback of the letters may be successfully [18].

### Strengths and limitations

Several studies examined the presence and correctness of the whole patient record by determination of missing components and the inadequacy of the components [6, 7, 16, 19]. Some of these studies linked this examination to patient characteristics [7, 16]. However there are no studies in which the relation of missing discharge letters alone and its components is linked to patient characteristics.

One of the strengths of our study is that reviewing physicians were heavily depended on documentation stored in the patient record and in the hospital administrative system. Despite the fact that we did a thorough in-hospital search for missing discharge letters, some letters may have been missed. However, discharge letters that are not stored on a regular place may not be found by other health care workers which can be a potential risk for adverse events.

Our study has several limitations. First, the presence and the quality of the discharge letter components was only judged in the second stage of the review and therefore in records with at least one positive screening criteria for an AE. The results might be biased if the presence and quality of the components is different between records with and without positive screening criteria. A record with adverse events and preventable adverse events may be subjected to a lower quality of care and a lower quality of record keeping and discharge procedure. However, not all records with positive criteria had an adverse event or preventable adverse event; only 16% of these records contain an adverse event. Therefore a positive screening criterion is not directly associated with a lower quality of care.

Another limitation is that we only recorded the date of sending the discharge letter. From a patient safety perspective it is very informative if and when the discharge letter was received by de general practitioner as he plays an important role in preventing unplanned readmissions. Also, we do not have information on adverse events detected after discharge that might be related to incompleteness, incorrectness or untimeliness of the discharge letter.

## Conclusion

In conclusion, nearly ten percent of the discharge letters are lacking in patient records in Dutch hospitals. Discharge letters that are available are often lacking certain components. In 70% of the unplanned readmissions, the discharge letter of the index admission was missing or incomplete at the time of readmission. Discharge letters are more likely to be missing in patients electively admitted to a hospital, with a shorter length of stay, with less comorbidity, and in readmissions. It also seems that if something undesirable happened during the admission, discharge letters are more frequent available. Interventions to improve the completeness of the discharge letters should be tailored for and carried out on department level. We hope this study increases awareness of the fact that a substantial group of patients may be at risk for an adverse event, e.g. a readmission as the discharge letter of their admissions are not available or missing information for a good continuity of care. Awareness of this problem can stimulate the writing of complete, correct and timely discharge letters.

## Abbreviations

AE: Adverse event
ICC: Intra class correlation
IQ: Interquartile
